# Immunological Hallmarks of Inflammatory Status in Vaso-Occlusive Crisis of Sickle Cell Anemia Patients

**DOI:** 10.3389/fimmu.2021.559925

**Published:** 2021-03-11

**Authors:** Alexander Leonardo Silva-Junior, Nadja Pinto Garcia, Evilázio Cunha Cardoso, Stephanny Dias, Andrea Monteiro Tarragô, Nelson Abrahim Fraiji, Matheus Souza Gomes, Laurence Rodrigues Amaral, Andréa Teixeira-Carvalho, Olindo Assis Martins-Filho, Erich Vinicius De Paula, Allyson Guimarães Costa, Adriana Malheiro

**Affiliations:** ^1^Programa de Pós-Graduação em Ciências Aplicadas à Hematologia, Universidade do Estado do Amazonas (UEA), Manaus, Brazil; ^2^Diretoria de Ensino e Pesquisa, Fundação Hospitalar de Hematologia e Hemoterapia do Amazonas (HEMOAM), Manaus, Brazil; ^3^Programa de Pós-Graduação em Imunologia Básica e Aplicada, Instituto de Ciências Biológicas, Universidade Federal do Amazonas (UFAM), Manaus, Brazil; ^4^Laboratório de Bioinformática e Análises Moleculares, Rede Multidisciplinar de Pesquisa, Ciência e Tecnologia, Universidade Federal de Uberlândia, Patos de Minas, Brazil; ^5^Grupo Integrado de Pesquisas em Biomarcadores de Diagnóstico e Monitoração, Centro de Pesquisas René Rachou, Fundação Oswaldo Cruz (FIOCRUZ), Belo Horizonte, Brazil; ^6^Escola de Ciências Médicas, Universidade de Campinas, Campinas, Brazil; ^7^Programa de Pós-Graduação em Medicina Tropical, Universidade do Estado do Amazonas (UEA), Manaus, Brazil; ^8^Instituto de Pesquisa Clínica Carlos Borborema, Fundação de Medicina Tropical Dr. Heitor Vieira Dourado (FMT-HVD), Manaus, Brazil; ^9^Escola de Enfermagem de Manaus, Universidade Federal do Amazonas (UFAM), Manaus, Brazil

**Keywords:** molecules, hemolytic anemia, Brazilian Amazon, immune profile, biomarkers, inflammation

## Abstract

Sickle Cell Anemia (SCA) is the most common genetic disorder around the world. The mutation in the β-globin gene is responsible for a higher hemolysis rate, with further involvement of immunological molecules, especially cytokines, chemokines, growth factors, and anaphylatoxins. These molecules are responsible for inducing and attracting immune cells into circulation, thus contributing to increases in leukocytes and other pro-inflammatory mediators, and can culminate in a vaso-occlusive crisis (VOC). This study aimed to characterize the levels of these molecules in SCA patients in different clinical conditions in order to identify potential hallmarks of inflammation in these patients. An analytical prospective study was conducted using the serum of SCA patients in steady-state (StSt; *n* = 27) and VOC (*n* = 22), along with 53 healthy donors (HD). Samples from the VOC group were obtained on admission and on discharge, in the convalescent phase (CV). Levels of chemokines (CXCL8, CXCL10, CL2, CLL3, CCL4, CL5, and CCL11), cytokines (IL-1β, IL-1ra, IL-2, IL-4, IL-5, IL-6, IL-7, IL-10, IL-12p70, IL-13, IL-17A, TNF-α, and IFN-γ) and growth factors (VEGF, FGFb, PDGF-BB, GM-CSF, and G-CSF) were measured using a Luminex assay, and anaphylatoxins (C3a, C4a, and C5a) were measured using Cytometric Bead Array. SCA patients in StSt showed a pro-inflammatory profile, and were indicated as being higher producers of CCL2, IL-1β, IL-12p70, IFN-γ, IL-17A, and GM-CSF, while VOC is highlighted by molecules IL-4 and IL-5, but also IL-2, IL-7, PDGF-BB, and G-CSF. PDGF-BB and IL-1ra seemed to be two important hallmarks for the acute-to-chronic stage, due to their significant decrease after crisis inflammation and statistical difference in VOC and CV groups. These molecules show higher levels and a strong correlation with other molecules in VOC. Furthermore, they remain at higher levels even after crisis recovery, which suggest their importance in the role of inflammation during crisis and participation in immune cell adhesion and activation. These results support a relevant role of cytokines, neutrophil and monocytes, since these may act as markers of VOC inflammation in SCA patients.

## Introduction

Sickle cell anemia (SCA) is the most prevalent hemoglobinopathy around the world and the most severe form of a set of genetic disorders that involve the β-globin gene (1, 2). It is caused by the homozygous form of a single mutation (adenine > thymine) on 17th nucleotide from region 15.5 of the long arm of chromosome 11, and results in production of a valine (instead of glutamic acid) and formation of a tetrameric protein known as hemoglobin S (HbS) (1, 3–7).

The mutation induces major alterations in the structure of red blood cells (RBC) secondary to the polymerization of HbS in areas of low oxygen tensions (8). These alterations lead to changes in the interaction of RBC with platelets, leukocytes and endothelial cells, contribute to both vaso-occlusion of small capillaries, and ischemia-reperfusion injury, and result in chronic hemolysis (1, 3). Accordingly, SCA is considered a chronic sterile inflammatory disease that occurs through ischemic injuries that contribute to the inflammatory process through the release of free hemoglobin during RBC hemolysis, besides other damage-associated molecular pattern (DAMP), such as heme and HMGB1. This leads to a stimulus of TLR4 and further promotes a chronic and sterile inflammation, adhesion of immune cells and vaso-occlusion process. The cellular response to this chronic stimulus contributes to the activation of neutrophils, monocytes, mast cells, endothelial cells, dendritic cells and NK cells, which are all regulated by levels of inflammatory mediator's that are driven mainly by immunological molecules (1, 8–14).

Although caused by a single mutation, the clinical presentation of SCA is modulated by the manner in which the immune system responds to chronic hemolysis and ischemia-reperfusion injury. Moreover, the disease is characterized by chronic progressive organ damage during periods known as steady-state (StSt), intercalated with acute episodes of vaso-occlusion, termed VOC, which are considered exacerbations of the pro-inflammatory condition of SCA with further formation of aggregates with immune cells, sickle RBCs and platelets (1, 8–10).

The aggregate rate is related to increase in the risk of VOC, and consequences of this include tissue injury, hypoxia, ischemia-reperfusion, renal dysfunction, acute chest syndrome, stroke, and finally, a decrease on the patient's life expectancy (3, 8, 10, 11, 15, 16). Even though many studies have analyzed immunological patterns in SCA (17–21), the relationship between these molecules and VOC inflammatory status and clinical presentation, there are still some knowledge gaps.

This study aimed to evaluate whether and to what point cytokines, chemokines, anaphylatoxins, and growth factors are hallmarks of inflammatory status for SCA patients in different clinical conditions treated at a hematological reference hospital in the Brazilian Amazon. We show here that even after clinical recovery from VOC, SCA patients still presented a higher concentration of pro-inflammatory mediators.

## Materials and Methods

### Ethics Statement

The present study was submitted to and approved by the Ethical Committee at Fundação Hospitalar de Hematologia e Hemoterapia do Amazonas (CEP-HEMOAM), via the processes #1.864.640 and #2.478.469. All participants enrolled in the present investigation read and signed the informed consent form in accordance with the Declaration of Helsinki and Resolution 466/2012 of the Brazilian National Health Council for research involving human subjects.

### Subjects and Samples

Whole blood samples were collected through venipuncture from 53 healthy donors (HD) that were eligible for blood donation and had no infectious or genetic disease. Samples were also collected from 27 patients with SCA in steady-state (StSt) condition (defined as the absence of clinical symptoms associated with VOC), who had not received a blood transfusion in the 90 days prior to recruitment, and had negative serology tests for HIV, HCV, HBV, HTLV and Syphilis. In addition, samples were also obtained from 22 patients with SCA in VOC (characterized by acute pain located at lumbar, hip, bone, articulation or abdominal with no other cause), which had been confirmed by health professionals at HEMOAM; the reference hospital in the Amazonas state for treatment of patients with hematological diseases. An additional blood sample was obtained from patients in the VOC group, in the period between the patients' discharge and their first outpatient visit, within 90 days from enrollment. These samples were identified as the convalescence (CV) group. Clinical and epidemiological data was obtained from medical records. In regards to treatment, the following medications were recorded: folic acid, hydroxyurea, analgesics, corticoids, and anti-inflammatory drugs for more than 1 year prior to sample collection.

From all healthy donors and patients, 8 ml of whole blood was collected and divided equally into EDTA (BD Vacutainer^®^ EDTA K2) tubes and Gel separator (Gel BD SST^®^ II Advance) tubes. Whole blood in EDTA tubes was used for acquisition of hematological data for red blood cells (RBCs), white blood cells (WBCs) and platelets, which were obtained using an automatic hematological counter (ADVIA 2120i, Siemens, USA) at HEMOAM. Using centrifugation, serum was obtained from the tubes with separator gel and was then stored at −80°C until further assays.

### Quantification of Immunological Molecules

Serum was used for quantifying chemokines (CXCL8, CXCL10, CCL2, CCL3, CCL4, CCL5, and CCL11), cytokines (IL-1β, IL-1ra, IL-2, IL-4, IL-5, IL-6, IL-7, IL-10, IL-12p70, IL-13, IL-17A, IFN-γ, and TNF-α) and growth factors [G-CSF, GM-CSF, PDGF-BB, VEGF, and FGF Basic (FGFb)], and was performed using the Luminex technique at Instituto René Rachou (FIOCRUZ-MG). The Bioplex-Pro Human Cytokine 27-Plex Kit (Bio-Rad, California, USA) was used following the manufacturer's instructions and protocol. Data acquisition and molecule levels were measured on a Luminex 200 System and Bioplex Manager Software, respectively, using the Five Parameters Logistic Regression, with results expressed in pg/ml. The detection limit of molecules is as follows: CXCL8 = 42,150 pg/ml; CXCL10 = 31,236 pg/ml; CCL2 = 24,282 pg/ml; CCL3 = 960 pg/ml; CCL4 = 11,233 pg/ml; PDGF-BB = 24,721 pg/ml, CCL5 = 16,533 pg/ml; CCL11 = 26,842; IL-1β = 8,608 pg/ml; IL-1ra = 91,661 pg/ml; IL-2 = 18,297 pg/ml; IL-4 = 4,789 pg/ml; IL-5 = 23,105 pg/ml; IL-6 = 37,680 pg/ml; IL-7 = 16,593 pg/ml; IL-10 = 35,170 pg/ml; IL-12p70 = 37,684 pg/ml; IL-13 = 8,090 pg/ml; IL-17A = 28,850 pg/ml; IFN-γ = 25,411 pg/ml; TNF-α = 64,803 pg/ml; FGFb = 16,046 pg/ml; G-CSF = 40,049 pg/ml; GM-CSF = 12,844 pg/ml; and VEGF = 29,464 pg/ml. Due to bead analysis issues, IL-9 and IL-15 levels could not be performed. In addition, quantification of anaphylatoxins C3a, C4a, and C5a were performed using EDTA plasma samples with the BD™ CBA (Cytometric Bead Array) Human Anaphylatoxin kit (BD^®^ Biosciences, San Diego, CA, USA). A FACSCanto II flow cytometer was used for sample acquisition. The analysis of the concentration of anaphylatoxin molecules was conducted using FCAP-Array software v.3 (Soft Flow Inc., USA). The detection limits are as follows: C3a = 0.45 pg/ml; C4a = 0.70 pg/ml; C5a = 1.15 pg/ml.

### Statistical Analysis

Data analysis and graphs were performed using GraphPad Prism v.5.0 software (San Diego, CA, USA). The Shapiro-Wilk normality test was conducted for analysis of normality distribution and acquisition of median and (25th and 75th). The epidemiological data was compared for the groups using the Chi-square test (χ^2^). The median of hematological parameters and molecule levels was used for comparison of HD, StSt, and VOC using the Kruskal-Wallis test, followed by Dunn's Multiple Comparison Test. For VOC and CV group comparison, the Wilcoxon matched pair test was used. A *p*-value of <0.05 was considered significant for all statistical tests.

### Signature of Immunological Molecules

The median of each molecule for HD, StSt and VOC groups was calculated, as previously described (22), and used as the cut-off point. This was expressed in pg/ml (CXCL8 = 2.64; PDGF-BB = 292.0; CCL3 = 0.96; CCL4 = 10.74; CCL2 = 9.07; CCL5 = 57.0; IL-1β = 1.12; IL-1ra = 29.11; TNF-α = 12.12; IL-6 = 1.12; IL-7 = 2.82; IL-12p70 = 2.40; IL-2 = 0.44; IFN-γ = 15.85; IL-4 = 0.53; IL-5 = 2.93; IL-13 = 0.70; IL-17A = 6.74; IL-10 = 5.20; CXCL10 = 69.68; VEGF = 9.08; GM-CSF = 7.81; G-CSF = 1.24; FGFb = 3.64; CCL11 = 23.14; C3a = 10.03; C4a = 7.61; C5a = 316.9). This value was employed to classify the patients for each group as being either “High” or “Low” molecule producers. The percentage value was obtained, and presented in a Venn diagram when higher than the 50th percentile, and obtained using a public website (http://bioinformatics.psb.ugent.be/webtools/Venn/).

### Immunological Hallmarks Network

The correlation analysis was conducted using Spearman test in GraphPad Prism v.5.0 software (San Diego, CA, USA), and employed all molecules and blood cell parameters for each group. The data was transferred to a spreadsheet (Microsoft Excel 2010), and the cytokine network was visualized on the open access software Cytoscape v.3.7.2. For all networks, each parameter was represented by a circular node, while a significant correlation was represented by a line connecting both correlated nodes. Absolute values of the correlation index (*r*) was used in order to classify correlation strength as weak (*r* < 0), moderate (*r* ≥ 0.36 and ≤ 0.68), or strong (*r* > 0.68), which is represented by line thickness, while positive and negative correlation was represented by continuous and dashed lines, respectively, as previously proposed (23).

### Heatmap and Decision Tree Analysis

The heatmap analyses were performed using the serum concentration levels of each biomarker evaluated using heatmap.2 function in R software (Project for Statistical Computing Version 3.0.1) and the gplots package. The decision trees were built using the WEKA software (Waikato Environment for Knowledge Analysis, version 3.6.11, University of Waikato, New Zealand) in order to classify SCA patients based on immunological markers. Leave-one-out-cross-validation (LOOCV) was applied in order to estimate the classification accuracy and to test the generalizability of the model.

## Results

### Epidemiological and Laboratorial Data

SCA patients presented a median of 22 years of age in StSt and VOC groups, while the median age of the control group was 30 years (*p* = 0.0324). Males were the majority gender in the HD group (70%), while females were the majority in the StSt group (67%). Data regarding place of residence and chronic pharmacological treatment is described in Table 1.

**Table 1:** Epidemiological data of HD and SCA patients, showing age, gender, city of residence, and chronic pharmacological treatment.

**Variable**	**HD *n* = 53**	**StSt *n* = 27**	**VOC *n* = 22**	**CV *n* = 22**	*****p***-value**
**Age (years, median [IQR])**	30 [23–42]	30 [22–34]	22 [14–34]	22 [14–34]	***0.032***
**Gender**, ***n (%)***					
Male	37 (70)	9 (37)	11 (50)	11 (50)	***0.016^a^***
Female	16 (30)	18 (67)	11 (50)	11 (50)	
**Place of residence**, ***n (%)***					
Manaus, AM	53 (100)	23 (85)	20 (91)	20 (91)	0.063
Interior of Amazonas state	–	4 (15)	2 (9)	2 (9)	
**Chronic pharmacological treatment**, ***n (%)***					
Folic acid	–	25 (93)	17 (77)	17 (77)	0.219
Hydroxyureia	–	22 (81)	14 (63)	14 (63)	0.202
Analgesic	–	12 (44)	12 (54)	12 (54)	0.570
Corticoid	–	2 (7)	–	–	1
Anti-inflammatory	–	1 (4)	5 (23)	5 (23)	0.077

*HD, Healthy Donors; StSt, Steady-state; VOC, Vaso-Occlusive Crisis; CV, Convalescence*.

a *Significant difference for HD vs. StSt*.

*Statistical analysis performed by Kruskal-Wallis with Dunn's Multiple Comparison Test for the variable of age in order to compare HD, and SS and VOC. In addition, Wilcoxon test was performed for VOC and CV comparison. Gender and city of residence was compared using a Chi-Square (χ^2^) test. For both analyses, p was considered significant when <0.05*.

*Bold-type font indicates statistical significance*.

Hematological values of each group, the medians and the results of the statistical analysis are described in Table 2. Patients in StSt had lower RBC, hemoglobin and hematocrit levels, when compared to the healthy donors. In addition, SCA patients (both StSt and VOC) showed increased levels of reticulocytes. The VOC group was marked by higher WBC counts, which seem to be driven by neutrophil and monocyte involvement, although only the neutrophil level was statistically lower after crisis. Basophil levels decreased in conditions of StSt to VOC, but no significant difference was observed in conditions of VOC and the convalescent phase. Even though platelet level had no statistical difference in SCA patients, it was higher than in the HD group. SCA patients, regardless of inflammatory status, showed higher involvement of lymphocytes and platelets.

**Table 2:** Laboratorial data of hematological parameters from HD, StSt, VOC and CV groups.

**Variables**	**HD *n* = 53**	**StSt *n* = 27**	**VOC *n* = 22**	**CV *n* = 22**	*****p***-value**
RBC (×10^6^/μL, median [IQR])	4.99[4.59–5.40]	2.51[2.19–2.75]	2.39[2.09–2.94]	2.65[2.29–3.12]	**<0.0001^a^^,^^b^**
Hemoglobin (g/dL, median [IQR])	14.90[13.55–15.95]	8.0[7.10–9.0]	7.50[6.20–8.45]	8.30[7.20–8.70]	**<0.0001^a^^,^^b^**
Hematocrit (%, median [IQR])	43.70[40.45–47.30]	24.50[21.70-28.50]	22.0[18.60–25.0]	24.80[21.95–26.68]	**<0.0001^a^^,^^b^^,^^d^**
MCV (fL, median [IQR])	87.80[84.85–90.4]	99.90[92.80–110.2]	91.30[79.60–99.80]	90.55[86.83–99.15]	**<0.0001^a^^,^^c^**
MCH (pg, median [IQR])	29.70[28.90–30.60]	32.10[30.30–36.70]	31.10[27.35–33.20]	29.70[27.58–34.03]	**0.0008^a^**
MCHC (g/dL, median [IQR])	34.10[33.10–34.60]	32.70[31.90–34.10]	33.60[32.90–34.05]	33.15[32.40–33.95]	**0.0057^a^**
RDW (fL, median [IQR])	13.70[12.95-13.95]	18.20[17.30–19.80]	21.20[19.65–23.80]	19.95[18.08–22.90]	**<0.0001^a^^,^^b^**
Reticulocyte (×10^3^/μL, median [IQR])	72.70[56.90–88.80]	367.6[226.0–529.9]	257.4[134.3–349.2]	248.6[134.5–332.8]	**<0.0001^a^^,^^b^**
WBC (×10^6^/μL, median [IQR])	6.33[5.27–7.10]	7.42[5.95–9.44]	11.39[8.82–15.17]	9.51[8.07–11.38]	**<0.0001^b^^,^^c^**
Neutrophil (x10^3^/μL, median [IQR])	3.43[2.80–4.18]	3.39[2.13–4.81]	6.90[4.87–9.46]	4.88[3.05–6.43]	**<0.0001^b^^,^^c^^,^^d^**
Lymphocyte (v10^3^/μL, median [IQR])	1.84[1.55–2.16]	2.41[2.13–3.36]	3.02[2.32–4.78]	3.28[2.43–4.96]	**<0.0001^a^^,^^b^**
Monocyte (×10^3^/μL, median [IQR])	0.38[0.29–0.41]	0.43[0.33–0.69]	0.66[0.52–0.86]	0.60[0.41–0.83]	**<0.0001^b^^,^^c^**
Basophil (×10^3^/μL, median [IQR])	0.03[0.02–0.05]	0.03[0.03–0.05]	0.00[0.00–0.03]	0.00[0.00–0.04]	**0.0002^b^^,^^c^**
Eosinophil (×10^3^/μL, median [IQR])	0.19[0.12–0.41]	0.24[0.13–0.50]	0.22[0.10–0.62]	0.42[0.24–0.62]	0.6031
Platelet count (×10^3^/μL, median [IQR])	244[213–281.5]	420[372–533]	411[326–530]	460.5[339.8–642.0]	**<0.0001^a^^,^^b^**

a *Significant difference for HD vs. StSt*.

b *Significant difference for HD vs. VOC*.

c *Significant difference for StSt vs. VOC*.

d *Significant difference for VOC vs. CV. Statistical analysis performed using Kruskal-Wallis with Dunn's Multiple Comparison Test in order to compare HD, StSt and VOC. In addition, Wilcoxon test was performed for VOC and CV comparison. For both analyses, p was considered significant when <0.05*.

*Bold-type font indicates statistical significance*.

### SCA Is Marked by an Inflammatory Molecule Profile Regardless of Clinical Condition

**Figure 1:**
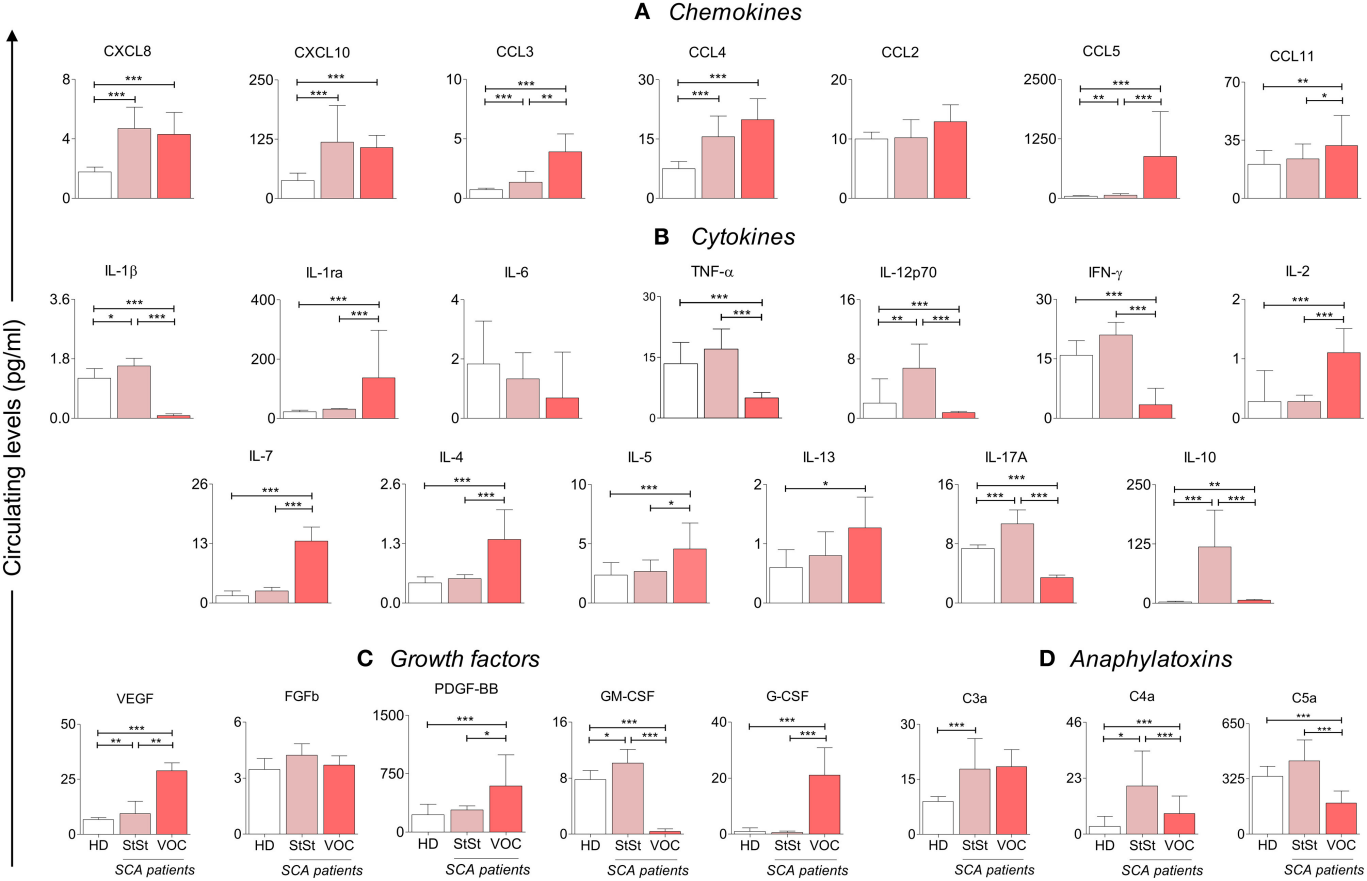
SCA patients in VOC (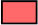) condition show an anti-inflammatory cytokine profile, when compared to StSt (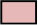) and HD (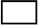) groups. Statistically significant values were considered when *p* < 0.05, and are presented as ^*^p < 0.05; ^**^p < 0.01; ^***^p < 0.001. Chemokines **(A)**, cytokines **(B)** and growth factors **(C)** were measured using Luminex, and anaphylatoxins **(D)** measured using CBA. Data is presented as median values and interquartile range in pg/ml. Statistical analysis was performed using Kruskal-Wallis and Dunn's Multiple Comparison Test. HD, healthy donors; StSt, Steady-state; VOC, vaso-occlusive crisis.

With the aim of characterizing the profile of serum biomarkers in SCA patients, a range of soluble mediators were quantified in StSt, VOC groups, and compared to the HD group. Significantly higher levels of chemokines (CXCL8, CXCL10, CCL3, CCL4, CCL5), cytokines (IL-1β, IL-12p70, IL-17A, IL-10), growth factors (VEGF and GM-CSF) and anaphylatoxin C4a were found in SCA patients, when compared to the HD group, as shown in Figure 1. Chemokines seemed to be more involved in VOC, when compared to StSt, through increased levels of CCL3, CCL5, and CCL11 (Figure 1A). Furthermore, IL-4 and IL-5 levels were higher (Figure 1B). The inflammatory status observed in the StSt group was characterized by an increased concentration of the pro-inflammatory molecules IL-1β, TNF-α, IL12p70, IFN-γ, and IL-17A, despite there being higher circulating levels of IL-10, GM-CSF, C4a, and C5a, when compared to the VOC group (Figures 1A,C,D). In addition, in our molecule analysis we observed that patients in the VOC group have significantly higher median values of cell proliferation markers.

### Signature of Immune Molecules Presented by HD, StSt, and VOC Groups

**Figure 2:**
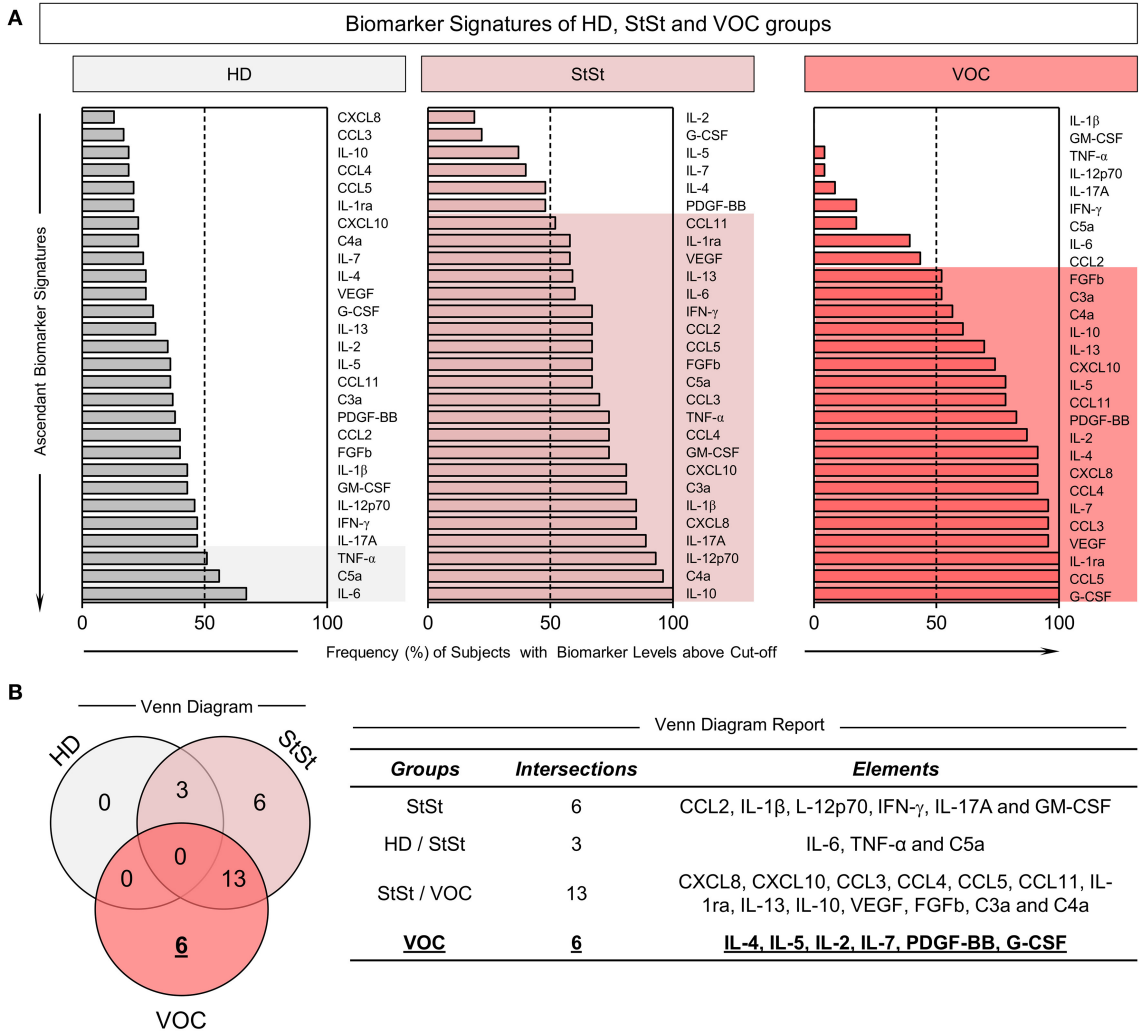
Immunological molecules in VOC clinical condition of SCA patients presented in a Venn diagram. **(A)** Ascendant Biomarker Signature of HD, StSt and VOC groups based on frequency of subjects with biomarker levels above Cut-off. **(B)** Venn Diagram with respective groups, intersections, and elements reported as potential hallmarks. The elements describe which molecules are potential hallmarks for each clinical condition and controls. Molecules were measured using Luminex and CBA. The global median for each soluble molecule was calculated and used as a cut-off point in order to classify groups as low (<50%) or high (>50%) producers of chemokines, cytokines, growth factors and anaphylatoxins. HD, healthy donors; StSt, steady-state; VOC, vaso-occlusive crisis.

Figure 2 summarizes the biomarker signatures and presents the Venn diagram of immunological molecules, with respective intersections and elements for the HD, StSt, and VOC groups. Our aim was to describe which group was classified as being the highest producer of molecules, and which belong exclusively to each group (Figure 2A). Inflammatory status in the StSt group showed that the majority of patients have increased levels of 22 soluble immune molecules, while the StSt group presented as higher producers of only six: CCL2, IL-1β, IL-12p70, IFN-γ, IL-17A, and GM-CSF, based on the majority of patients and the global median (Figure 2B). Our analysis did not identify any molecule that all three groups share as high producers, however, the HD and StSt groups both showed higher production of TNF-α, C5a, and IL-6, and SCA patients, regardless of inflammatory status presented themselves as higher producers of 13 immune molecules (Figure 2B). Although 19 molecules were identified as having higher production in VOC, only six were shown exclusively in this stage: IL-2, IL-7, IL-4, IL-5, PDGF-BB, and G-CSF, which suggests that the VOC condition is orchestrated not just by anti-inflammatory cytokines, but also by intense cell proliferation (Figure 2B).

### Potential of Immunological Markers IL-1β, IL-10, IL-1ra, and IL-6 for Distinguishing Clinical Conditions (StSt, VOC, and CV) in SCA Patients

**Figure 3:**
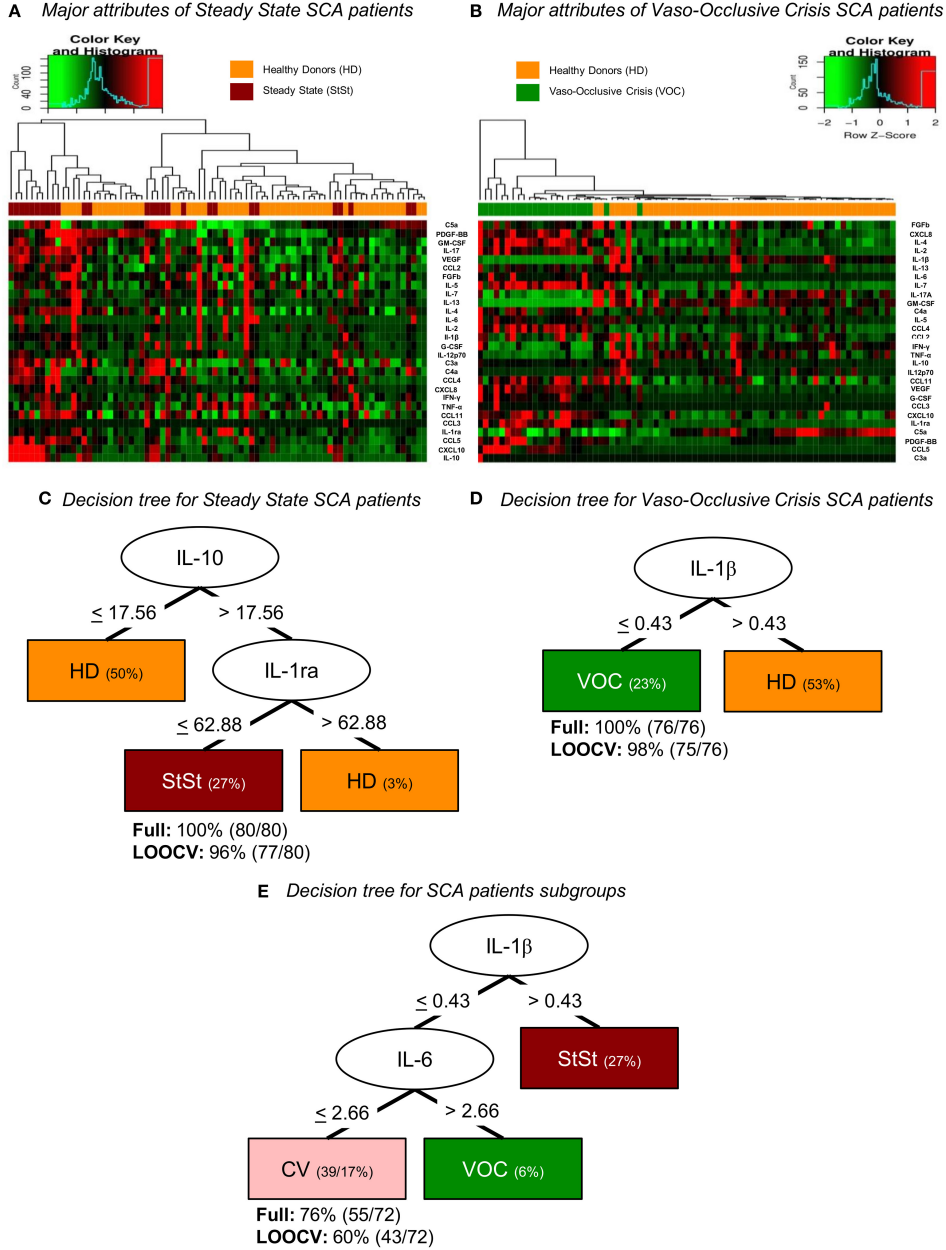
Bioinformatic analysis of serum molecules divided into the attributes of the control group and SCA subgroups according to clinical condition, represented by heatmaps **(A,B)** and decision trees **(C–E)** of z-score normalized events. **(A)** Molecule attributes showed the ability to cluster healthy individuals and steady-state SCA patients. **(B)** Heatmap analysis also shows high ability to distinguish controls and vaso-occlusive SCA patients. **(C)** Decision tree analysis provides the clustering based on IL-10 circulating levels in order to classify individuals as HD if ≤ 17.56 pg/ml or if > 17.56 pg/ml, analyze IL-1ra level to categorize as HD if > 62.88 pg/ml or as StSt if ≤ 62.88 pg/ml. **(D)** Decision tree analysis provides clustering of HD and VOC groups based on IL-1β circulating levels in order to categorize individuals as HD if > 0.43 pg/ml or as VOC if ≤ 0.43 pg/ml. **(E)** Decision tree analysis provides clustering of SCA patients based on IL-1β circulating levels in order to classify individuals as StSt if > 0.43 pg/ml or if ≤ 0.43 pg/ml, analyze IL-6 level to categorize as VOC if > 2.66 pg/ml or as CV if ≤ 2.66 pg/ml. HD, healthy donors; StSt, steady-state; VOC, Vaso-occlusive crisis; CV, Convalescence; LOOCV, Leave-One-Out Cross Validation.

The heatmap analysis was performed with the serum levels of the immune molecules of SCA patients to demonstrate the components used in the clustering of the StSt or VOC subgroups when compared to the healthy individuals. Even though histograms of the comparison of the HD (top bar yellow) group with the SCA StSt (top bar red) group (Figure 3A) and the VOC (top bar green) group (Figure 3B) had better clustering when compared to SCA patients, our tree decision analysis for SCA subgroups highlighted IL-10 and IL-1ra levels as the major attributes for characterizing healthy individuals and patients in StSt based on molecule profile, with a global accuracy of 100%, which reached 96% after LOOCV (Figure 3C). Analysis showed that circulating levels of IL-10 when ≤ 17.56 pg/ml indicated an HD group, while when >17.56 pg/ml, a further analysis contributed to identify HD if IL-1ra >62.88 pg/ml or StSt if IL-1ra ≤ 62.88 pg/ml (Figure 3C). In order to characterize the HD and VOC groups, the serum biomarker levels contributed to cluster the HD group if IL-1β >0.43 pg/ml or VOC if IL-1β ≤ 0.43 pg/ml with 100% accuracy, which reached 98% after LOOCV (Figure 3D). Under SCA subgroups, IL-1β can also be used to categorize StSt patients, when >0.43 pg/ml, or if <0.43 pg/ml, a further analysis contribute to identify VOC if IL-6 >2.66 pg/ml or CV if IL-6 ≤ 2.66 pg/ml (Figure 3E).

### Hallmarks of Immune Molecules in Acute-to-Chronic SCA Patients After VOC

**Figure 4:**
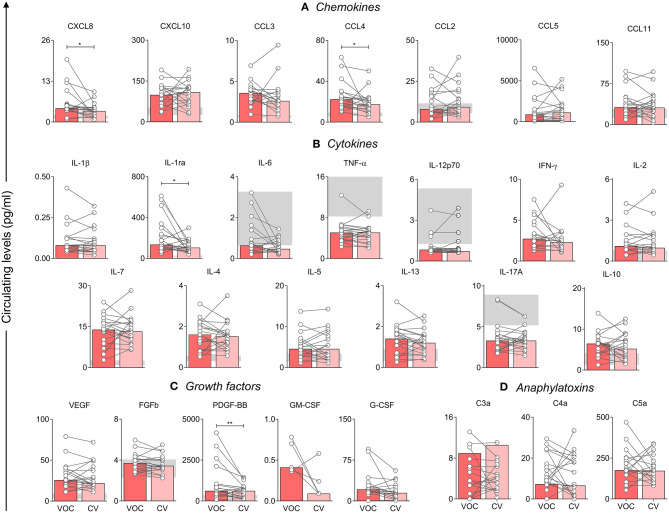
Analysis of the first molecules that decrease after crisis, when compared with VOC (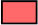) and CV (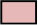) groups. Statistical analysis was performed using the Wilcoxon test. A *p*-value < 0.05 was considered statistically significant and was presented as ^*^p < 0.05; ^**^p < 0.01. Chemokines **(A)**, cytokines **(B)**, and growth factors **(C)** were measured using Luminex, and anaphylatoxins **(D)** using CBA. Data is shown as median values and interquartile range, using data in pg/ml. VOC, Vaso-occlusive crisis; CV, Convalescence.

A follow-up was performed in patients during the VOC condition, which compared samples obtained on admission and after convalescence. By analyzing the results obtained, we could identify the most sensitive markers of SCA physiopathology after VOC recovery. The median time between sample collection was 53 days. However, our results show that the inflammatory profile did not change significantly. Both the VOC and the CV states maintained a similar immunological profile, with the exception of CXCL8, CCL4, IL-1ra, and PDGF-BB (Figures 4A–C). Even though CXCL8 and CCL4 had significantly lower levels in CV, there was no observed difference in median values in StSt and VOC groups, which was different from IL-1ra and PDGF-BB. As shown in Figures 1, 4, hallmarks of acute-to-chronic transition can be marked by all four of these molecules, although only IL-1ra and PDGF-BB showed significant differences in both states.

### SCA Patients Display a Complex Correlation Network With Different Involvement in Immune Molecules Based on Inflammatory Status

**Figure 5:**
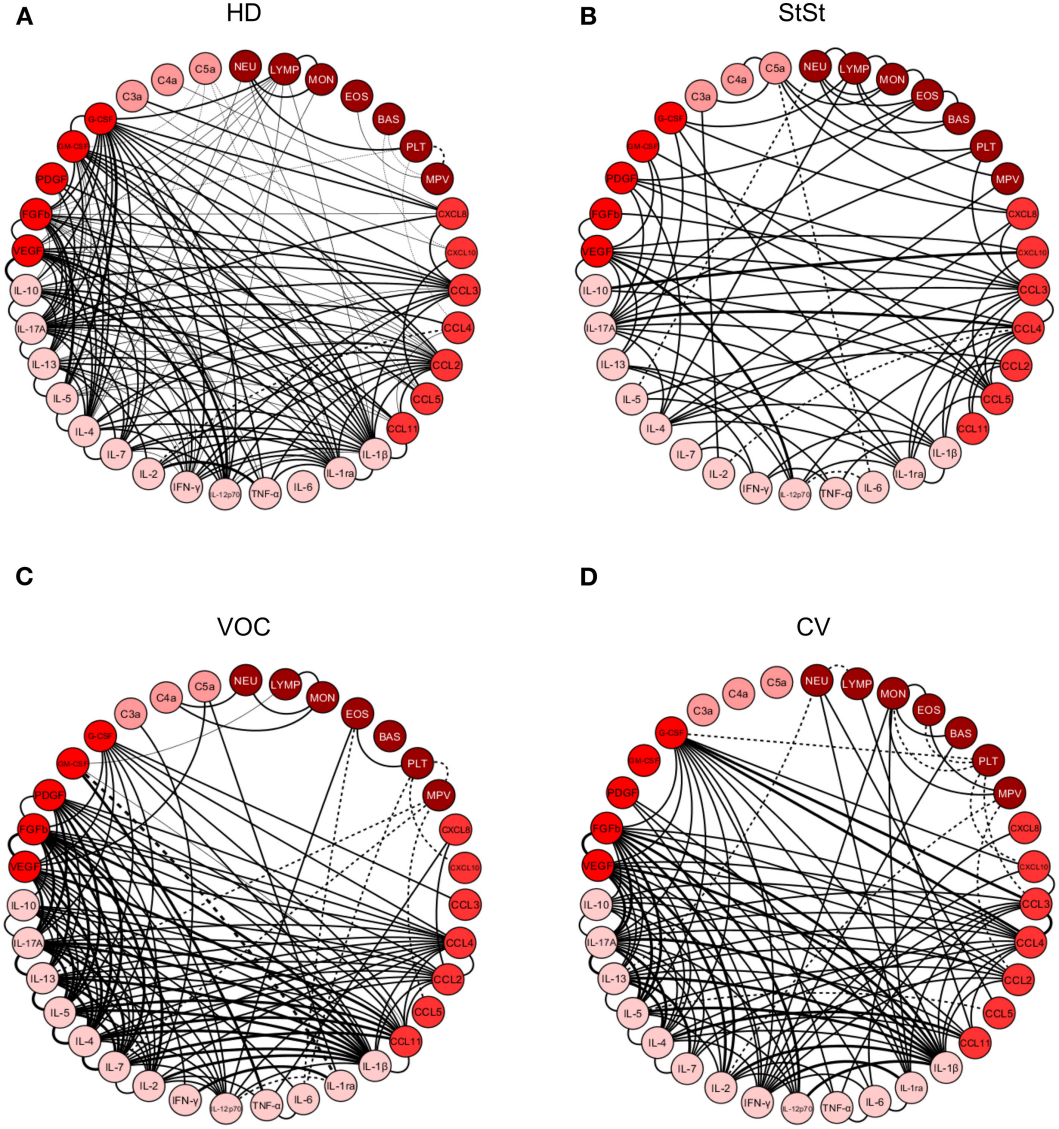
Correlation analysis presented as a network of immunological cytokines, chemokines, growth factors, anaphylatoxins, and leukocytes in healthy donors **(A)**, steady-state **(B)** vaso-occlusive crisis **(C)**, and convalescence **(D)** stages. Each parameter is shown in a node. Statistical analysis was performed using the Spearman correlation test and the significant correlations (*p* < 0.05) are represented by a line connecting both nodes. The correlation was classified as weak (*r* < 0), moderate (0.36 < *r* < 0.68) and strong (*r* > 0.68), based on absolute value of correlation index *r*, represented by line thickness. Positive correlation is expressed by a continuous line, while negative correlation by dashed lines. NEU, Neutrophil; LYMP, Lymphocytes; MON, Monocytes; EOS, Eosinophils; BAS, Basophils; PLT, Platelets; MPV, Mean platelet volume; chemokines (CXCL8; CXCL10; CCL3; CCL4; CCL2; CCL5; CCL11), cytokines (IL-1β; IL-1ra; IL-6; TNF-α; IL-12p70; IFN-γ; IL-2; IL-7; IL-4; IL-5; IL-13; IL-17A; IL-10), growth factors (VEGF; FGFb; PDGF; GM-CSF; G-CSF) and anaphylatoxins (C3a; C4a; C5a). HD, Healthy donors; StSt, Steady-state; VOC, vaso-occlusive crisis; CV, convalescence.

Despite the function of most immune molecules being already known, correlation analysis allows us to observe interaction among the groups. Thus, we observed that correlation analysis in the HD group and the SCA StSt and VOC patients had different patterns. While StSt patients have less interactions, a chronic inflammatory condition mediated by monocyte, driven by IL-17A, IL-12p70, CXCL10, and CCL4 (Figure 5B) can be seen when compared to the HD group (Figure 5A). In contrast, the VOC group had an increase in correlation molecules, which was highlighted by the inflammatory pattern and polarized to an anti-inflammatory response, and showed a main interaction of IL-1β, IL-2, IL-7, IL-4, IL-5, IL-13, IL-17A, FGFb, and GM-CSF (Figure 5C). Besides the strong and positive correlations observed, GM-CSF and IL-1ra had a strong but negative correlation. The CV group network had a lower correlation index (Figure 5D), although the molecules described in VOC still seemed to have higher participation in the acute-to-chronic inflammation process.

**Figure 6:**
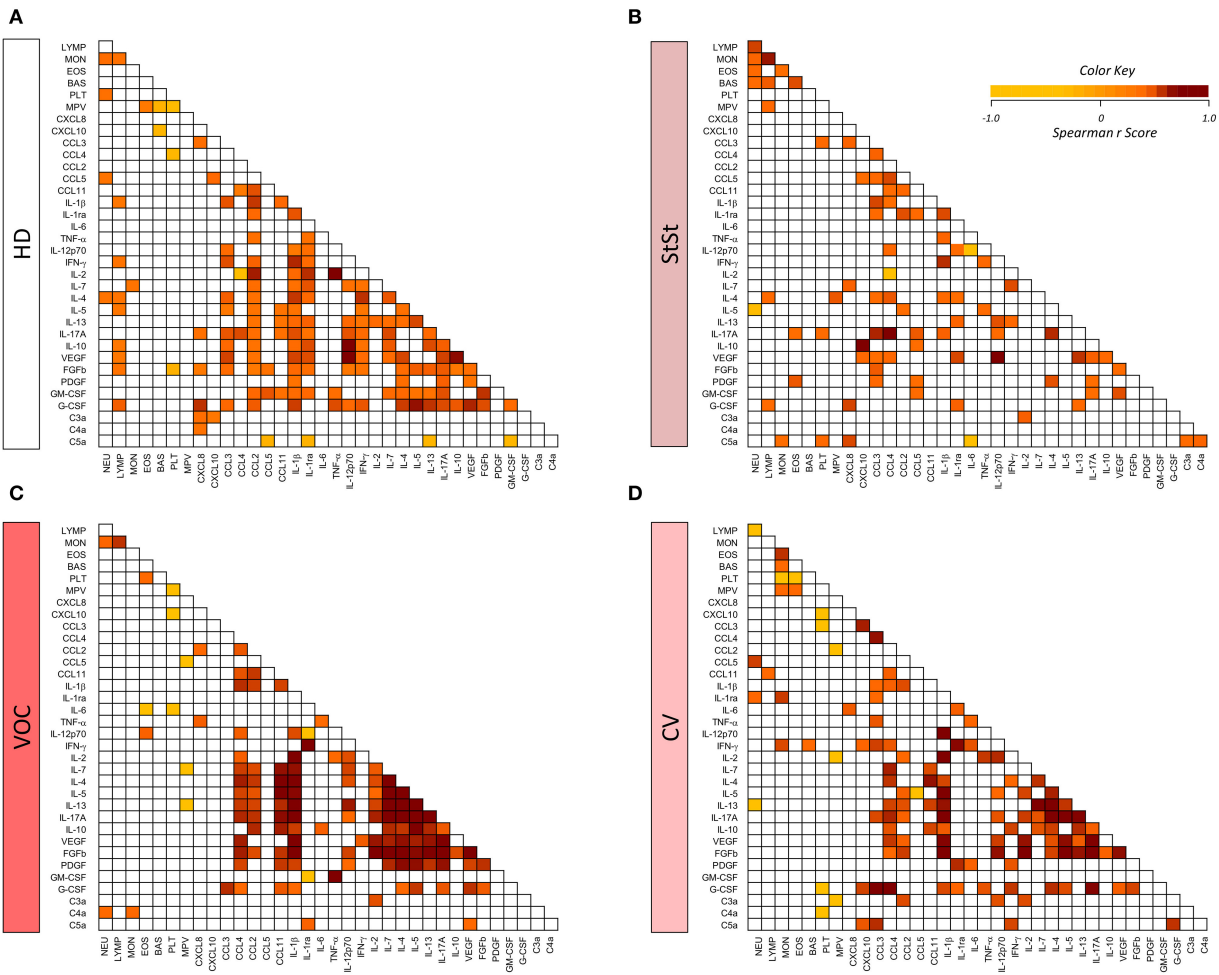
Biomarker correlation matrices illustrate distinct patterns of biomarker connectivity in healthy donors **(A)**, steady-state **(B)**, vaso-occlusive crisis **(C)** and convalescence **(D)** stages. Biomarker networks were based on the Spearman's correlation indices (*r*). Correlation matrices display significant association (*p* < 0.05) between biomarker pairs based on the rank indices, which are tagged by color keys, ranging from −1.0 to 1.0 to underscore the correlation strength, according to the color key provided in the figure. NEU, Neutrophil; LYMP, Lymphocytes; MON, Monocytes; EOS, Eosinophils; BAS, Basophils; PLT, Platelets; MPV, Mean platelet volume; chemokines (CXCL8; CXCL10; CCL3; CCL4; CCL2; CCL5; CCL11), cytokines ((IL-1β; IL-1ra; IL-6; TNF-α; IL-12p70; IFN-γ; IL-2; IL-7; IL-4; IL-5; IL-13; IL-17A; IL-10), growth factors (VEGF; FGFb; PDGF; GM-CSF; G-CSF), and anaphylatoxins (C3a; C4a; C5a). HD, healthy donors; StSt, steady-state; VOC, vaso-occlusive crisis; CV, convalescence.

Correlation matrices further corroborate these findings and highlight that while HD (Figure 6A) displayed a hallmark network with an overall moderate connectivity and the StSt group (Figure 6B) presented a panoramic network with less neighborhood connections, the VOC patients exhibited a higher level of immune marker connectivity, particularly within the cytokine axis (Figure 6C). As the VOC group shift toward the CV group (Figure 6D) a clear downregulation of biomarker connectivity can be observed.

## Discussion

SCA is marked by intense inflammation that is secondary to systemic injury and clinical status. The inflammatory process is evidenced by several interactions of cells, such as neutrophils, monocytes, platelets, and RBCs, which are involved in the pathogenesis of this condition. Accordingly, immunological molecules, especially cytokines, chemokines, growth factors, and anaphylatoxins are also relevant as regulators of this process. Although several studies have evaluated the levels of these molecules and their association with clinical characteristics of SCA, little evidence is available regarding the interaction of these molecules in the individuals with SCA, particularly during VOC and in the transition from the acute-to-chronic state after a VOC. The main finding of our study was the ability to use IL-1β, IL-10, and IL-1ra levels to segregate subgroups of SCA patients.

Patients in StSt have less disease severity and show no threatening clinical symptoms, in comparison to those patients in crisis. Even with no severe symptoms, inflammatory markers are still present, in comparison to healthy controls. In addition, these molecules are involved in immune response that contributes to vaso-occlusion episodes (3, 8, 24). The chronic inflammation in StSt seem to be characterized by increased levels in the pro-inflammatory cytokines IL-1β, TNF-α, IL-12p70, IFN-γ, and circulating cells but with less endothelial involvement, similarly to what has been observed in other studies (18, 25–28). It has already been established that neutrophils, monocytes and pro-inflammatory molecules, together with platelets, play an important role in disease severity (3, 9, 26, 29). Increased levels of IL-10 in the StSt has been described as part of T-cell differentiation (30), VOC development and disease severity (31), suggesting that this cytokine participates in the process of regulating the pro-inflammatory state. Furthermore, other factors, such as infectious or other genetic diseases, influence inflammatory response and contribute to vaso-occlusion, thus, reducing patient's life expectancy (1, 10).

Acute inflammation is characterized by local ischemia/reperfusion injury, leukocyte recruitment and circulating cell activation, which contribute to severe clinical symptoms in a VOC (3, 8). Some studies describe the participation of anti-inflammatory cytokines in this condition (8, 20, 24, 30) and, in addition, our results show that it is marked mainly by IL-1ra and IL-4 molecules, with involvement of adherent neutrophils and monocytes. Many studies have focused on differences in molecule levels in StSt and VOC (17–19, 32), However few studies have focused on immunological hallmarks, which can be used to describe the transition between inflammatory states (StSt, VOC, and convalescence). Increased levels of IL-2 and IL-7, together with growth factors, have been previously observed (20) and contribute to proliferation and maturation of granulocytes. In addition, the findings regarding chemokines support the statement that these circulating cells show a higher capacity to adhere to endothelial cells and form cell-to-cell and cell-endothelium aggregates in VOC, which contributes to endothelial injury, inflammatory marker production, immune cell recruitment, vaso-occlusion and consequently severe clinical complications, as described by other authors (3, 10, 29, 30, 33). Our data demonstrated that VOC patients displayed a lack of canonical pro-inflammatory factors and a clear increase in regulatory mediators. This may suggest that VOC is not an anti-inflammatory condition *per se*, but it may be linked to a skew in the “type” of inflammation rather than its magnitude/strength. The analysis of biomarker networks and the correlation matrices between pairs of soluble mediators demonstrated that there is strong connectivity between pro-inflammatory/regulatory cytokines in VOC. The strength of IL-1β connections in VOC was noteworthy and may suggest that the inflammasome activation may participate in SCA pathophysiology.

We identified that the alternative pathway of the complement system is not that different under StSt or VOC, contrary to what has been described by other authors. IL-1β and IL-17A might be indirectly related to classical activation of the complement system through C-reactive protein (CRP) production from the liver (32, 34–36), and further interaction with natural antibodies (37), culminating in higher C4 cleavage rate. Free heme interacts with C1q ligands (CRP and immunoglobulin) and leads to less classical complement activation in VOC (38); and with C3, culminating on higher C3 cleavage rate (39, 40). Free heme availability and its direct and indirect interaction to complement molecules may explain why the SCA groups had no significant difference in C3a levels. This increased activation of the complement pathway has already been described in StSt patients and observed in our results (41). Little information related to the involvement of the complement system in SCA is available, but even though the production of anaphylatoxins is well defined, the function of anaphylatoxins as inflammatory or regulatory molecules remains unclear in SCA pathophysiology.

Surprisingly, both HD and StSt groups were identified as higher producers of the pro-inflammatory molecules TNF-α and IL-6, although only TNF-α levels were significantly higher in VOC, though not IL-6, as observed in some studies (18, 27, 28) and in contrast to others (17, 20, 32, 36, 42–44). VOC was characterized as being a higher producer of anti-inflammatory and immune cell proliferation cytokines. It is important to notice that IL-4 and IL-5 are also produced by activated mast cells, in which have been reported during VOC in mice, and are important contributor on pain (14). Even though higher levels of some cytokines have been described by other authors, this characterization has never been described before for SCA patients.

Complementarily, our bioinformatic analysis permitted the segregation of SCA patients based on circulating IL-1β, IL-10, IL-1ra, and IL-6 levels, and regardless of their role, we described these molecules as potential hallmarks for segregating these patients into StSt, VOC, and CV groups. The decision trees show novel proposals for biomarkers that should to be investigated in further studies for a better comprehension of SCA physiopathology and thus may contribute to better clinical decisions.

The CV group was an intermediary period in VOC and StSt stages, for which we observed that the first inflammatory mediators to decrease were CXCL8, CCL4, IL-1ra, and PDGF-BB after hospital release after a VOC episode. However, only IL-1ra and PDGF-BB presented statistical differences in VOC and StSt groups. Even though IL-1ra is known to be an anti-inflammatory marker, its concentration was related to increased events of pain (45). As such, we believe that these results may contribute to the SCA patient's follow-up after treatment for VOC episodes. In addition, the correlations allowed us to identify that, during this acute-to-chronic transition, some interactions in the main molecules responsible for cell proliferation still remain, which indicates that there still is stimulus for leukocytosis, even though the inflammation pattern does not differ that much from VOC.

A strong and positive correlation under TNF-α and GM-CSF in VOC was identified in our analysis, which sustains a positive inflammatory pattern, with further leukocyte recruitment and activation, especially neutrophils and monocytes (9, 33). Negative feedback is observed in both StSt and VOC conditions, the first is mediated by IL-10, while the second by IL-1ra, which is an inactive antagonist of IL-1β. This statement is supported by the IL-10/CXCL10 and IL-1ra/GM-CSF axis in StSt and VOC, respectively. IL-10's role as a biomarker in SCA is still controversial, since some authors describe lower levels in StSt, when compared to the control group, together with CXCL10 (43), while others found increased levels (20, 30, 46, 47) and some show no difference (17).

The present study has some limitations. Since SCA is considered a sterile inflammatory disease, the assessment of the TLRs expression, as well as the analysis of checkpoints in immune cell subsets along with quantification of other cytokines (IL-1a, IL-18, and IL-33), would provide a more detailed description regarding the inflammasome activation in order to more fully understand SCA pathophysiology and allow for the identification of novel prognostic factors. These aspects remain to be elucidated in future investigations.

Our study brought new perspectives for inflammatory knowledge of SCA. In fact, the role of many molecules in SCA is still discussable whether inflammatory or regulatory, as well as their association to a VOC development or as a consequence of a VOC.

## Conclusion

Herein, we highlight the interactions of IL-4 and IL-2 cytokines in VOC, as well as the efficacy of IL-1ra and PDGF-BB as markers of clinical recovery post-VOC. In addition, we describe the ability of IL-10 and IL-1ra levels to cluster patients into HD or StSt, and IL-1β levels to cluster patients into HD or VOC. Our results contribute to novel markers in the Brazilian Amazon SCA population, and suggest their potential in prognosis and follow-up after hospital recovery from VOC. The present study is the first report on inflammatory hallmarks in VOC and CV in sickle cell anemia patients and supports greater understanding of disease pathophysiology mechanisms in order to identify novel inflammatory biomarkers and contribute to therapeutic perspectives.

## Data Availability Statement

The original contributions presented in the study are included in the article/supplementary material, further inquiries can be directed to the corresponding author/s.

## Ethics Statement

The studies involving human participants were reviewed and approved by the Ethical Committee at Fundação Hospitalar de Hematologia e Hemoterapia do Amazonas (CEP-HEMOAM), via the processes #1.864.640 and #2.478.469. All participants enrolled in the present investigation read and signed the informed consent form in accordance with the Declaration of Helsinki and Resolution 466/2012 of the Brazilian National Health Council for research involving human subjects. The patients/participants provided their written informed consent to participate in this study.

## Author Contributions

AS-J, AC, and AM designed, performed the experiments, analyzed data, and wrote the manuscript. AS-J, MG, LA, OM-F, and AC analyzed data. AS-J, NG, EC, SD, and AT recruited all individuals, performed the experiments, and revised the manuscript. NF, AT-C, and ED revised the manuscript. AS-J, NG, AT, OM-F, AT-C, and AM supervised the project development, designed the experiments, interpreted the data, wrote, and revised the manuscript. All authors read and approved the final manuscript.

## Conflict of Interest

The authors declare that the research was conducted in the absence of any commercial or financial relationships that could be construed as a potential conflict of interest.
